# Crystal structure of 2-cyano-1-methyl­pyridinium tetra­fluoro­borate

**DOI:** 10.1107/S2056989015016011

**Published:** 2015-09-12

**Authors:** Francesca A. Vaccaro, Lynn V. Koplitz, Joel T. Mague

**Affiliations:** a Department of Chemistry, Loyola University, New Orleans, LA 70118, USA; b Department of Chemistry, Tulane University, New Orleans, LA 70118, USA

**Keywords:** crystal structure, salt, C—H⋯F inter­actions

## Abstract

The asymmetric unit of the title salt, C_7_H_7_N_2_^+^·BF_4_^−^, comprises two independent but nearly identical formula units. The solid-state structure comprises corrugated layers of cations and anions, formed by C—H⋯F hydrogen bonding, that are approximately parallel to (010). Further C—H⋯F hydrogen bonding consolidates the three-dimensional architecture. The sample was refined as a two-component non-merohedral twin.

## Related literature   

For structures of other salts of the 2-cyano-1-methyl­pyridinium cation, see: Koplitz *et al.* (2012 ▸); Kammer *et al.* (2013 ▸). For structures of salts of the isomeric 2-cyano­anilinium cation, see: Zhang (2009 ▸); Cui & Chen (2010 ▸). 
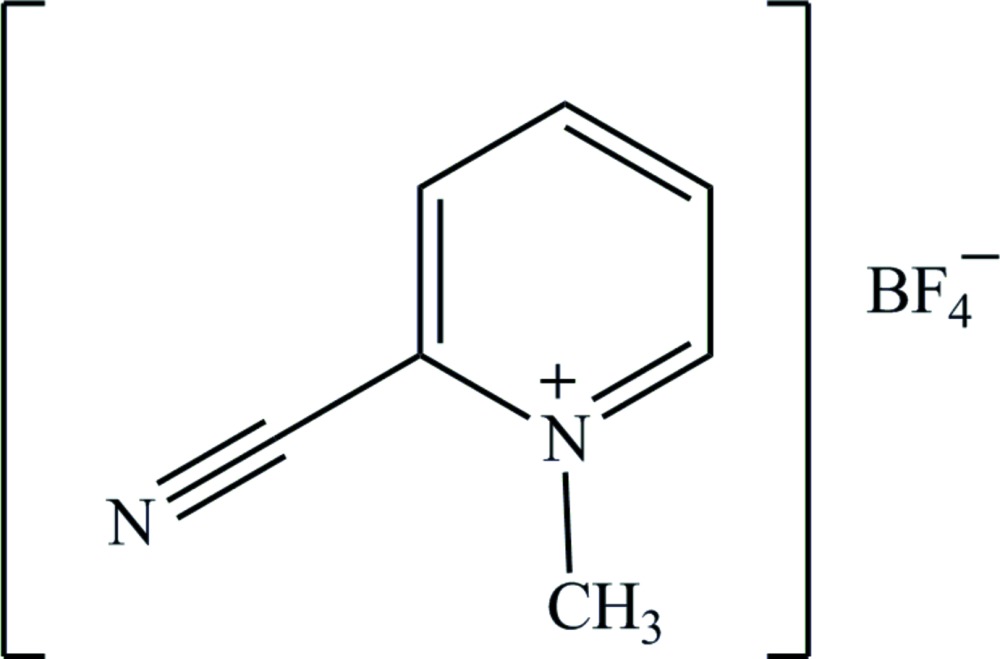


## Experimental   

### Crystal data  

C_7_H_7_N_2_^+^·BF_4_^−^
*M* *_r_* = 205.96
Monoclinic, 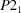

*a* = 7.9704 (16) Å
*b* = 7.5527 (15) Å
*c* = 14.570 (3) Å
β = 90.312 (3)°
*V* = 877.1 (3) Å^3^
*Z* = 4
Mo *K*α radiation
μ = 0.15 mm^−1^
*T* = 150 K
0.14 × 0.13 × 0.08 mm

### Data collection  

Bruker SMART APEX CCD diffractometer
Absorption correction: multi-scan (*SADABS*; Bruker, 2014 ▸) *T*_min_ = 0.70, *T*_max_ = 0.99
16120 measured reflections
4566 independent reflections
3779 reflections with *I* > 2σ(*I*)
*R*_int_ = 0.057

### Refinement  

*R*[*F* ^2^ > 2σ(*F* ^2^)] = 0.050
*wR*(*F* ^2^) = 0.122
*S* = 1.08
4566 reflections
256 parameters
1 restraint
H-atom parameters constrained
Δρ_max_ = 0.33 e Å^−3^
Δρ_min_ = −0.27 e Å^−3^
Absolute structure: the absolute structure could not be determined with certainty in this light-atom structure

### 

Data collection: *APEX2* (Bruker, 2014 ▸); cell refinement: *SAINT* (Bruker, 2014 ▸); data reduction: *SAINT*; program(s) used to solve structure: *SHELXT* (Sheldrick, 2015*a* ▸); program(s) used to refine structure: *SHELXL2014* (Sheldrick, 2015*b* ▸); molecular graphics: *DIAMOND* (Brandenburg & Putz, 2012 ▸); software used to prepare material for publication: *SHELXTL* (Sheldrick, 2008 ▸) and *PLATON* (Spek, 2009 ▸).

## Supplementary Material

Crystal structure: contains datablock(s) global, I. DOI: 10.1107/S2056989015016011/tk5380sup1.cif

Structure factors: contains datablock(s) I. DOI: 10.1107/S2056989015016011/tk5380Isup2.hkl

Supporting information file. DOI: 10.1107/S2056989015016011/tk5380Isup3.cml

**Fig. 1.**
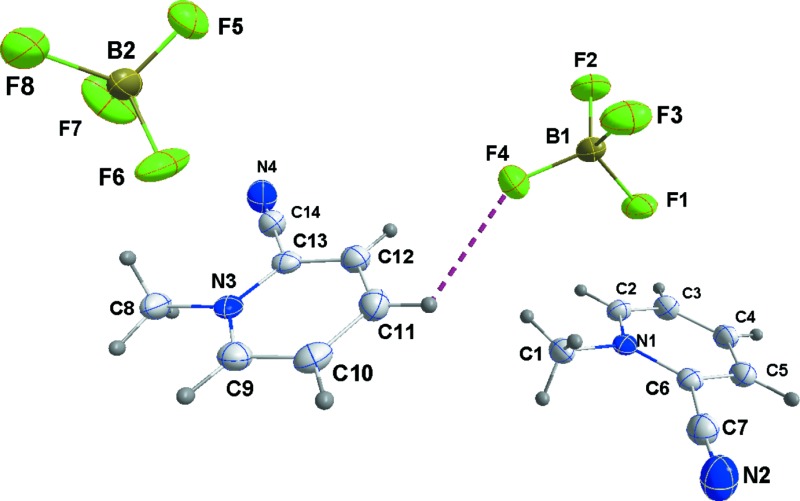
Perspective view of the asymmetric unit with 50% probability ellipsoids. The C—H···F interaction is shown by a dotted line.

**Fig. 2.**
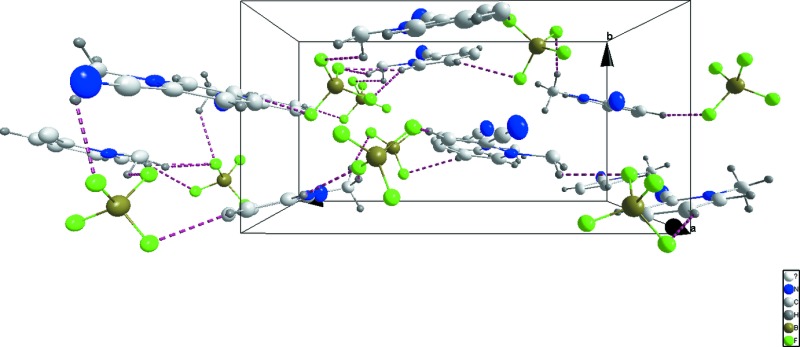
Packing viewed down the a axis showing an edge view of two corrugated layers and the C—H···F interactions (dotted lines) holding them together.

**Fig. 3.**
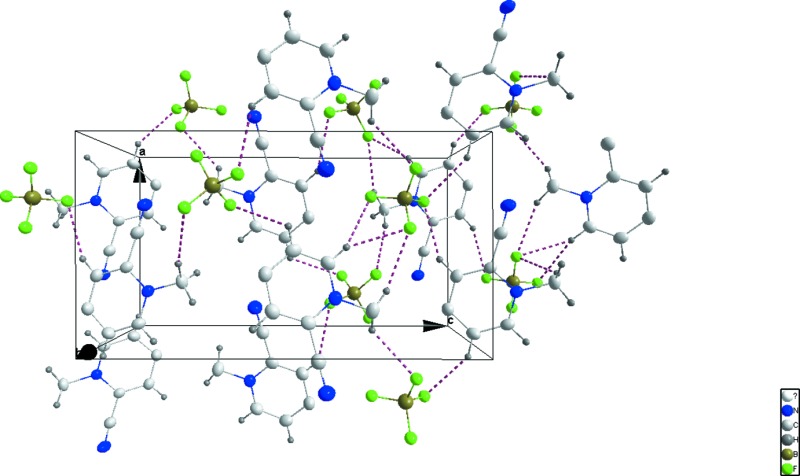
Packing viewed down the b axis providing a plan view of the corrugated sheets with C—H···F interactions shown as dotted lines.

. DOI: 10.1107/S2056989015016011/tk5380fig1.tif
Perspective view of the asymmetric unit with 50% probability ellipsoids. The C—H⋯F inter­action is shown by a dotted line.

a . DOI: 10.1107/S2056989015016011/tk5380fig2.tif
Packing viewed down the *a* axis showing an edge view of two corrugated layers and the C—H⋯F inter­actions (dotted lines) holding them together.

b . DOI: 10.1107/S2056989015016011/tk5380fig3.tif
Packing viewed down the *b* axis providing a plan view of the corrugated sheets with C—H⋯F inter­actions shown as dotted lines.

CCDC reference: 1420782

Additional supporting information:  crystallographic information; 3D view; checkCIF report

## Figures and Tables

**Table 1:** Hydrogen-bond geometry (, )

*D*H*A*	*D*H	H*A*	*D* *A*	*D*H*A*
C1H1*A*F7^i^	0.98	2.50	3.407(6)	154
C1H1*B*F8^ii^	0.98	2.54	3.498(6)	166
C1H1*C*F3^iii^	0.98	2.47	3.214(5)	132
C2H2F7^i^	0.95	2.29	3.190(5)	157
C3H3F1^iv^	0.95	2.46	3.294(6)	147
C5H5F1^v^	0.95	2.45	3.306(5)	149
C8H8*A*F2^i^	0.98	2.48	3.159(6)	126
C8H8*C*F3^ii^	0.98	2.55	3.437(6)	151
C9H9F3^ii^	0.95	2.52	3.392(6)	152
C9H9F4^ii^	0.95	2.59	3.476(6)	156
C10H10F6^ii^	0.95	2.54	3.167(6)	123
C12H12F5^i^	0.95	2.49	3.277(6)	141

Symmetry codes: (i) 
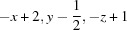
; (ii) 
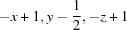
; (iii) 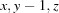; (iv) 
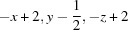
; (v) 
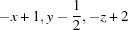
.

## supplementary crystallographic information

### S1. Comment

The asymmetric unit consists of two independent formula units. A portion of the C—H···F hydrogen bonding network which aids the packing of the several ions is shown in Fig. 1 with fuller depictions appearing in Figs 2 and 3. The solid state structure comprises corrugated layers of cations and anions formed by C—H···F hydrogen bonding between them and approximately parallel to (010). These layers are held to one another by additional C—H···F interactions.

### S2. Experimental

To 0.64 g (0.5 mmol) of 2-cyano-1-methylpyridinium iodide dissolved in 8.5 ml of 95% ethanol was added 1.08 g (0.55 mmol) of solid silver tetrafluoroborate with stirring. The reaction mixture was filtered to remove the precipitated AgI and the filtrate allowed to evaporate to dryness. From the resulting mass, crystals suitable for X-ray diffraction were selected.

### S3. Refinement

The H-atoms attached to carbon were placed in calculated positions (C—H = 0.95 - 0.98 Å). All were included as riding contributions with isotropic displacement parameters 1.2 - 1.5 times those of the attached atoms. In the late stages of the refinement a consistent pattern of F_o_^2^ >> F_c_^2^ suggested twinning not yet accounted for. Use of the *TwinRotMat* routine in *PLATON* (Spek, 2009) generated the twin law -1 0 0 0 - 1 0 0 0 1, inclusion of which enabled satisfactory refinement as a 2-component twin.

### Crystal data

**Table:**

C_7_H_7_N_2_^+^·BF_4_^−^	*F*(000) = 416
*M**_r_* = 205.96	*D*_x_ = 1.560 Mg m^−^^3^
Monoclinic, *P*2_1_	Mo *K*α radiation, λ = 0.71073 Å
*a* = 7.9704 (16) Å	Cell parameters from 8457 reflections
*b* = 7.5527 (15) Å	θ = 2.6–29.0°
*c* = 14.570 (3) Å	µ = 0.15 mm^−^^1^
β = 90.312 (3)°	*T* = 150 K
*V* = 877.1 (3) Å^3^	Block, colourless
*Z* = 4	0.14 × 0.13 × 0.08 mm

### Data collection

**Table:**

Bruker SMART APEX CCD diffractometer	4566 independent reflections
Radiation source: fine-focus sealed tube	3779 reflections with *I* > 2σ(*I*)
Graphite monochromator	*R*_int_ = 0.057
Detector resolution: 8.3660 pixels mm^-1^	θ_max_ = 29.3°, θ_min_ = 2.6°
φ and ω scans	*h* = −10→10
Absorption correction: multi-scan (*SADABS*; Bruker, 2014)	*k* = −10→10
*T*_min_ = 0.70, *T*_max_ = 0.99	*l* = −19→19
16120 measured reflections	

### Refinement

**Table:**

Refinement on *F*^2^	Secondary atom site location: difference Fourier map
Least-squares matrix: full	Hydrogen site location: inferred from neighbouring sites
*R*[*F*^2^ > 2σ(*F*^2^)] = 0.050	H-atom parameters constrained
*wR*(*F*^2^) = 0.122	*w* = 1/[σ^2^(*F*_o_^2^) + (0.0572*P*)^2^ + 0.091*P*] where *P* = (*F*_o_^2^ + 2*F*_c_^2^)/3
*S* = 1.08	(Δ/σ)_max_ < 0.001
4566 reflections	Δρ_max_ = 0.33 e Å^−^^3^
256 parameters	Δρ_min_ = −0.27 e Å^−^^3^
1 restraint	Absolute structure: The absolute structure could not be determined with certainty in this light-atom structure
Primary atom site location: structure-invariant direct methods	

### Special details

**Table:**

Experimental. The diffraction data were obtained from 3 sets of 400 frames, each of width 0.5° in ω, collected at φ = 0.00, 90.00 and 180.00° and 2 sets of 800 frames, each of width 0.45° in φ, collected at ω = -30.00 and 210.00°. The scan time was 10 sec/frame.
Geometry. All e.s.d.'s (except the e.s.d. in the dihedral angle between two l.s. planes) are estimated using the full covariance matrix. The cell e.s.d.'s are taken into account individually in the estimation of e.s.d.'s in distances, angles and torsion angles; correlations between e.s.d.'s in cell parameters are only used when they are defined by crystal symmetry. An approximate (isotropic) treatment of cell e.s.d.'s is used for estimating e.s.d.'s involving l.s. planes.
Refinement. Refinement of *F*^2^ against ALL reflections. The weighted *R*-factor *wR* and goodness of fit *S* are based on *F*^2^, conventional *R*-factors *R* are based on *F*, with *F* set to zero for negative *F*^2^. The threshold expression of *F*^2^ > σ(*F*^2^) is used only for calculating *R*-factors(gt) *etc*. and is not relevant to the choice of reflections for refinement. *R*-factors based on *F*^2^ are statistically about twice as large as those based on *F*, and *R*- factors based on ALL data will be even larger. H-atoms attached to carbon were placed in calculated positions (C—H = 0.95 - 0.98 Å). All were included as riding contributions with isotropic displacement parameters 1.2 - 1.5 times those of the attached atoms. In the late stages of the refinement a consistent pattern of *F*_o_^2^ >> *F*_c_^2^ suggested twinning not yet accounted for. Use of the *TwinRotMat* routine in *PLATON* (Spek, 2009) generated the twin law -1 0 0 0 - 1 0 0 0 1, inclusion of which enabled satisfactory refinement as a 2-component twin.

### Fractional atomic coordinates and isotropic or equivalent isotropic displacement parameters (Å^2^)

**Table:**

	*x*	*y*	*z*	*U*_iso_*/*U*_eq_	
N1	0.7369 (5)	0.1294 (4)	0.8819 (3)	0.0220 (7)	
N2	0.3067 (5)	0.0851 (7)	0.8963 (3)	0.0416 (11)	
C1	0.6868 (6)	0.1642 (6)	0.7854 (3)	0.0273 (10)	
H1A	0.7826	0.2126	0.7518	0.041*	
H1B	0.5943	0.2497	0.7841	0.041*	
H1C	0.6504	0.0534	0.7565	0.041*	
C2	0.8990 (6)	0.1331 (6)	0.9067 (3)	0.0264 (9)	
H2	0.9807	0.1629	0.8620	0.032*	
C3	0.9511 (6)	0.0952 (7)	0.9947 (3)	0.0299 (10)	
H3	1.0671	0.0973	1.0100	0.036*	
C4	0.8341 (6)	0.0543 (6)	1.0601 (3)	0.0283 (10)	
H4	0.8678	0.0281	1.1213	0.034*	
C5	0.6652 (6)	0.0520 (6)	1.0352 (3)	0.0274 (9)	
H5	0.5819	0.0254	1.0795	0.033*	
C6	0.6204 (5)	0.0884 (6)	0.9464 (3)	0.0232 (8)	
C7	0.4472 (6)	0.0856 (7)	0.9165 (3)	0.0295 (10)	
B1	0.7236 (7)	0.6589 (6)	0.8140 (3)	0.0242 (10)	
F1	0.7151 (4)	0.5195 (3)	0.8770 (2)	0.0328 (6)	
F2	0.8600 (4)	0.7654 (4)	0.8350 (2)	0.0361 (7)	
F3	0.5771 (3)	0.7603 (4)	0.8200 (2)	0.0389 (7)	
F4	0.7353 (4)	0.5925 (4)	0.72573 (19)	0.0446 (8)	
N3	0.7497 (5)	0.3325 (5)	0.3758 (2)	0.0246 (8)	
N4	1.1617 (6)	0.4550 (8)	0.3990 (3)	0.0454 (12)	
C8	0.8099 (6)	0.3192 (7)	0.2807 (3)	0.0316 (10)	
H8A	0.9110	0.2455	0.2790	0.047*	
H8B	0.8361	0.4378	0.2575	0.047*	
H8C	0.7225	0.2655	0.2421	0.047*	
C9	0.5886 (6)	0.2988 (6)	0.3939 (3)	0.0292 (10)	
H9	0.5148	0.2630	0.3460	0.035*	
C10	0.5294 (6)	0.3159 (6)	0.4825 (3)	0.0302 (10)	
H10	0.4148	0.2926	0.4954	0.036*	
C11	0.6365 (6)	0.3668 (7)	0.5519 (3)	0.0310 (10)	
H11	0.5962	0.3775	0.6129	0.037*	
C12	0.8039 (6)	0.4028 (6)	0.5327 (3)	0.0304 (10)	
H12	0.8794	0.4387	0.5798	0.036*	
C13	0.8566 (6)	0.3847 (6)	0.4439 (3)	0.0255 (9)	
C14	1.0267 (7)	0.4226 (7)	0.4190 (3)	0.0327 (10)	
B2	0.7732 (7)	0.8421 (7)	0.3142 (3)	0.0273 (10)	
F5	0.8153 (4)	0.9543 (4)	0.3856 (2)	0.0435 (8)	
F6	0.6889 (5)	0.6960 (4)	0.3476 (2)	0.0500 (9)	
F7	0.9153 (4)	0.7890 (6)	0.2691 (2)	0.0569 (10)	
F8	0.6664 (4)	0.9294 (4)	0.2535 (2)	0.0464 (8)	

### Atomic displacement parameters (Å^2^)

**Table:**

	*U*^11^	*U*^22^	*U*^33^	*U*^12^	*U*^13^	*U*^23^
N1	0.0256 (18)	0.0142 (16)	0.0263 (17)	0.0013 (14)	0.0025 (15)	−0.0004 (14)
N2	0.029 (2)	0.059 (3)	0.038 (2)	0.004 (2)	0.0041 (18)	−0.002 (2)
C1	0.037 (3)	0.022 (2)	0.023 (2)	−0.0010 (18)	−0.0026 (18)	0.0022 (17)
C2	0.023 (2)	0.023 (2)	0.033 (2)	−0.0011 (16)	0.0046 (19)	−0.0008 (19)
C3	0.025 (2)	0.033 (2)	0.032 (2)	0.0022 (19)	−0.0025 (19)	−0.002 (2)
C4	0.031 (2)	0.029 (2)	0.025 (2)	0.0000 (18)	−0.0006 (18)	−0.0012 (18)
C5	0.031 (2)	0.024 (2)	0.027 (2)	0.0002 (18)	0.0039 (19)	−0.0005 (18)
C6	0.023 (2)	0.0174 (19)	0.029 (2)	0.0007 (16)	0.0041 (17)	−0.0020 (17)
C7	0.029 (2)	0.028 (2)	0.032 (2)	0.0002 (18)	0.0046 (19)	−0.001 (2)
B1	0.029 (2)	0.018 (2)	0.026 (2)	−0.0007 (19)	0.001 (2)	0.0018 (18)
F1	0.0440 (15)	0.0189 (12)	0.0355 (14)	−0.0006 (12)	−0.0028 (13)	0.0047 (10)
F2	0.0336 (15)	0.0254 (14)	0.0492 (17)	−0.0069 (12)	−0.0028 (13)	0.0005 (12)
F3	0.0311 (15)	0.0235 (13)	0.062 (2)	0.0040 (12)	0.0021 (14)	0.0036 (13)
F4	0.069 (2)	0.0343 (16)	0.0304 (14)	−0.0020 (16)	0.0062 (15)	−0.0067 (13)
N3	0.0326 (19)	0.0141 (15)	0.0270 (18)	0.0007 (14)	0.0007 (15)	−0.0004 (14)
N4	0.034 (2)	0.060 (3)	0.042 (2)	−0.008 (2)	0.000 (2)	0.005 (2)
C8	0.041 (3)	0.025 (2)	0.028 (2)	−0.004 (2)	0.004 (2)	0.0001 (19)
C9	0.031 (2)	0.023 (2)	0.034 (2)	−0.0023 (17)	−0.005 (2)	0.0003 (19)
C10	0.029 (2)	0.023 (2)	0.039 (3)	−0.0003 (18)	0.0034 (19)	0.005 (2)
C11	0.037 (2)	0.029 (2)	0.027 (2)	0.0017 (19)	0.001 (2)	0.0019 (19)
C12	0.034 (2)	0.026 (2)	0.032 (2)	0.000 (2)	−0.003 (2)	0.0016 (19)
C13	0.029 (2)	0.0147 (18)	0.033 (2)	0.0023 (16)	−0.0026 (19)	0.0026 (17)
C14	0.035 (3)	0.031 (2)	0.032 (2)	−0.001 (2)	−0.002 (2)	0.002 (2)
B2	0.033 (3)	0.023 (2)	0.026 (2)	−0.002 (2)	0.002 (2)	0.004 (2)
F5	0.061 (2)	0.0328 (16)	0.0363 (16)	0.0103 (14)	−0.0076 (16)	−0.0078 (13)
F6	0.072 (2)	0.0196 (14)	0.059 (2)	0.0019 (15)	0.0198 (17)	0.0098 (14)
F7	0.0376 (17)	0.072 (3)	0.061 (2)	0.0042 (17)	0.0157 (15)	−0.0251 (19)
F8	0.057 (2)	0.0294 (16)	0.0522 (18)	−0.0021 (14)	−0.0197 (17)	0.0099 (14)

### Geometric parameters (Å, º)

**Table:**

N1—C2	1.340 (6)	N3—C9	1.336 (6)
N1—C6	1.360 (6)	N3—C13	1.362 (6)
N1—C1	1.484 (6)	N3—C8	1.473 (6)
N2—C7	1.157 (6)	N4—C14	1.143 (7)
C1—H1A	0.9800	C8—H8A	0.9800
C1—H1B	0.9800	C8—H8B	0.9800
C1—H1C	0.9800	C8—H8C	0.9800
C2—C3	1.376 (7)	C9—C10	1.382 (7)
C2—H2	0.9500	C9—H9	0.9500
C3—C4	1.372 (7)	C10—C11	1.375 (7)
C3—H3	0.9500	C10—H10	0.9500
C4—C5	1.392 (6)	C11—C12	1.392 (7)
C4—H4	0.9500	C11—H11	0.9500
C5—C6	1.369 (6)	C12—C13	1.370 (6)
C5—H5	0.9500	C12—H12	0.9500
C6—C7	1.446 (6)	C13—C14	1.434 (7)
B1—F4	1.384 (6)	B2—F7	1.372 (6)
B1—F2	1.385 (6)	B2—F6	1.382 (6)
B1—F1	1.398 (5)	B2—F5	1.382 (6)
B1—F3	1.400 (6)	B2—F8	1.391 (6)
			
C2—N1—C6	118.7 (4)	C9—N3—C13	120.6 (4)
C2—N1—C1	120.4 (4)	C9—N3—C8	119.4 (4)
C6—N1—C1	120.9 (4)	C13—N3—C8	119.9 (4)
N1—C1—H1A	109.5	N3—C8—H8A	109.5
N1—C1—H1B	109.5	N3—C8—H8B	109.5
H1A—C1—H1B	109.5	H8A—C8—H8B	109.5
N1—C1—H1C	109.5	N3—C8—H8C	109.5
H1A—C1—H1C	109.5	H8A—C8—H8C	109.5
H1B—C1—H1C	109.5	H8B—C8—H8C	109.5
N1—C2—C3	122.1 (4)	N3—C9—C10	119.9 (5)
N1—C2—H2	118.9	N3—C9—H9	120.0
C3—C2—H2	118.9	C10—C9—H9	120.0
C4—C3—C2	119.5 (4)	C11—C10—C9	120.0 (4)
C4—C3—H3	120.3	C11—C10—H10	120.0
C2—C3—H3	120.3	C9—C10—H10	120.0
C3—C4—C5	118.8 (4)	C10—C11—C12	119.9 (4)
C3—C4—H4	120.6	C10—C11—H11	120.0
C5—C4—H4	120.6	C12—C11—H11	120.0
C6—C5—C4	119.4 (4)	C13—C12—C11	118.0 (5)
C6—C5—H5	120.3	C13—C12—H12	121.0
C4—C5—H5	120.3	C11—C12—H12	121.0
N1—C6—C5	121.5 (4)	N3—C13—C12	121.5 (4)
N1—C6—C7	116.7 (4)	N3—C13—C14	117.6 (4)
C5—C6—C7	121.7 (4)	C12—C13—C14	120.9 (5)
N2—C7—C6	177.1 (5)	N4—C14—C13	179.1 (6)
F4—B1—F2	111.1 (4)	F7—B2—F6	109.9 (4)
F4—B1—F1	109.9 (4)	F7—B2—F5	110.0 (4)
F2—B1—F1	109.5 (4)	F6—B2—F5	110.0 (4)
F4—B1—F3	108.5 (4)	F7—B2—F8	109.7 (4)
F2—B1—F3	108.8 (4)	F6—B2—F8	107.8 (4)
F1—B1—F3	109.1 (4)	F5—B2—F8	109.5 (4)
			
C6—N1—C2—C3	0.7 (7)	C13—N3—C9—C10	−0.4 (7)
C1—N1—C2—C3	−177.6 (4)	C8—N3—C9—C10	−178.0 (4)
N1—C2—C3—C4	−0.9 (8)	N3—C9—C10—C11	−0.3 (7)
C2—C3—C4—C5	0.2 (8)	C9—C10—C11—C12	0.7 (8)
C3—C4—C5—C6	0.7 (7)	C10—C11—C12—C13	−0.3 (7)
C2—N1—C6—C5	0.2 (6)	C9—N3—C13—C12	0.7 (7)
C1—N1—C6—C5	178.5 (4)	C8—N3—C13—C12	178.4 (4)
C2—N1—C6—C7	−179.9 (4)	C9—N3—C13—C14	−178.6 (4)
C1—N1—C6—C7	−1.6 (6)	C8—N3—C13—C14	−0.9 (7)
C4—C5—C6—N1	−0.9 (7)	C11—C12—C13—N3	−0.3 (7)
C4—C5—C6—C7	179.2 (5)	C11—C12—C13—C14	178.9 (5)

### Hydrogen-bond geometry (Å, º)

**Table:**

*D*—H···*A*	*D*—H	H···*A*	*D*···*A*	*D*—H···*A*
C1—H1*A*···F7^i^	0.98	2.50	3.407 (6)	154
C1—H1*B*···F8^ii^	0.98	2.54	3.498 (6)	166
C1—H1*C*···F3^iii^	0.98	2.47	3.214 (5)	132
C2—H2···F7^i^	0.95	2.29	3.190 (5)	157
C3—H3···F1^iv^	0.95	2.46	3.294 (6)	147
C5—H5···F1^v^	0.95	2.45	3.306 (5)	149
C8—H8*A*···F2^i^	0.98	2.48	3.159 (6)	126
C8—H8*C*···F3^ii^	0.98	2.55	3.437 (6)	151
C9—H9···F3^ii^	0.95	2.52	3.392 (6)	152
C9—H9···F4^ii^	0.95	2.59	3.476 (6)	156
C10—H10···F6^ii^	0.95	2.54	3.167 (6)	123
C12—H12···F5^i^	0.95	2.49	3.277 (6)	141

Symmetry codes: (i) −*x*+2, *y*−1/2, −*z*+1; (ii) −*x*+1, *y*−1/2, −*z*+1; (iii) *x*, *y*−1, *z*; (iv) −*x*+2, *y*−1/2, −*z*+2; (v) −*x*+1, *y*−1/2, −*z*+2.
