# Supplementary figures and images for: Crystal structure of 2-cyano-1-methyl­pyridinium tetra­fluoro­borate
Source: Acta Crystallogr E Crystallogr Commun. 2015 Sep 12;71(Pt 10):o697–8. doi: 10.1107/S2056989015016011 (PMC4647419; doi:10.1107/S2056989015016011)

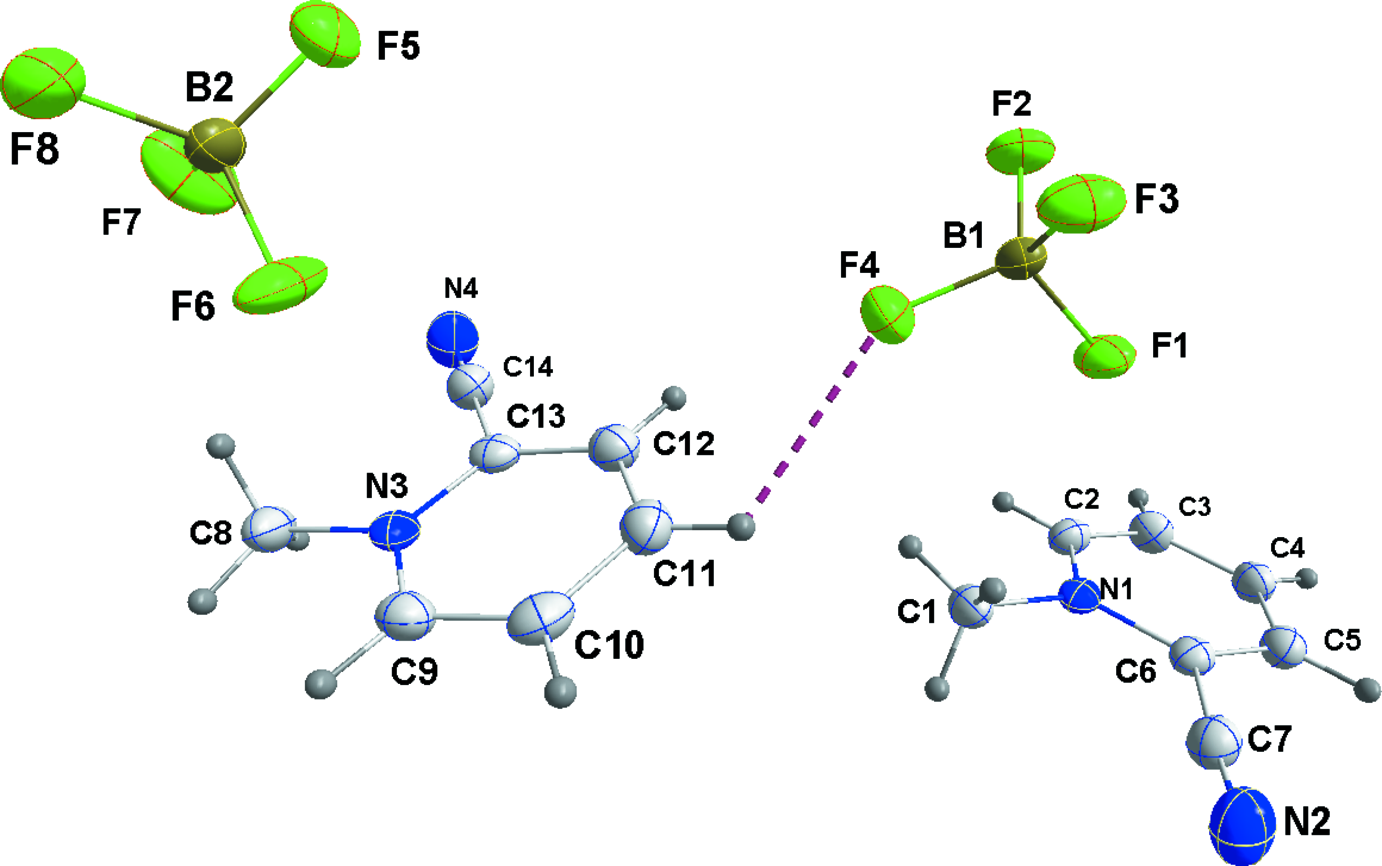

Supplement: Supplementary file 4 [file e-71-0o697-fig1.tif]

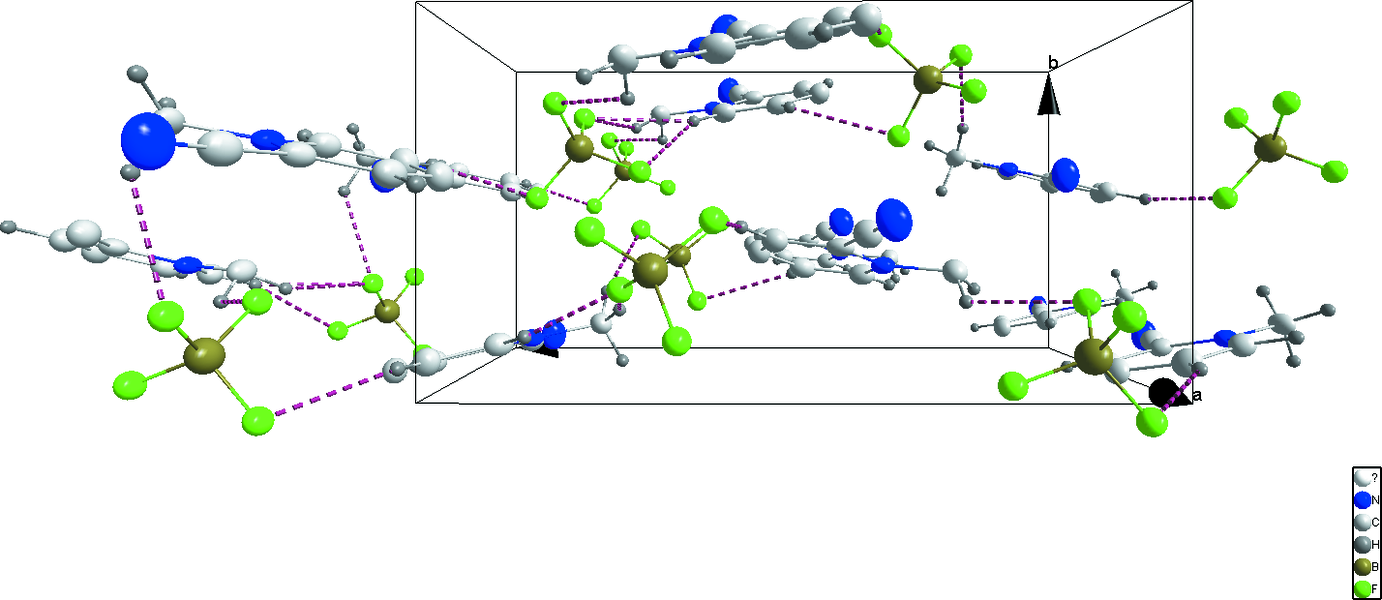

Supplement: Supplementary file 5 [file e-71-0o697-fig2.tif]

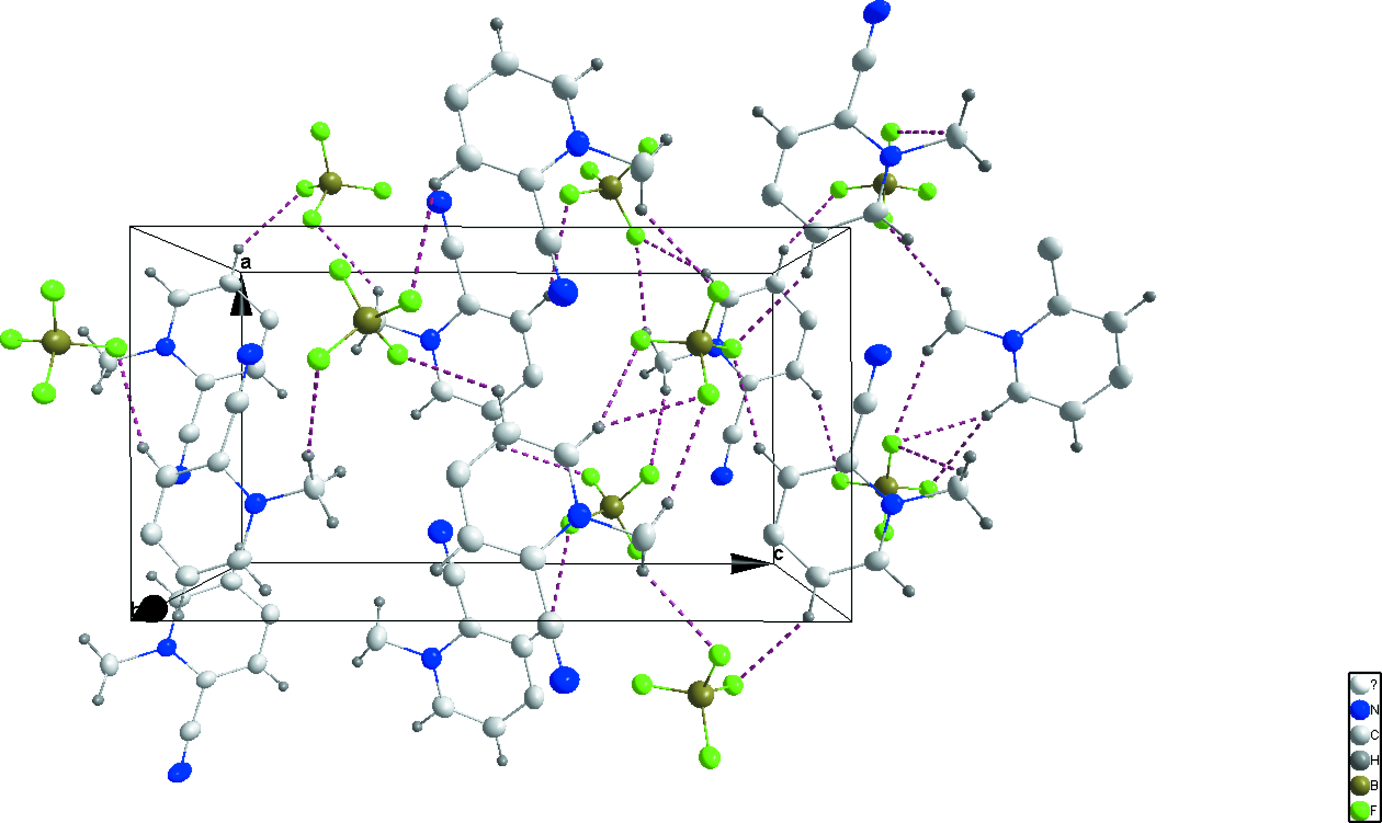

Supplement: Supplementary file 6 [file e-71-0o697-fig3.tif]
